# Cell Cycle–Specified Fluctuation of Nucleosome Occupancy at Gene Promoters

**DOI:** 10.1371/journal.pgen.0020158

**Published:** 2006-09-22

**Authors:** Gregory J Hogan, Cheol-Koo Lee, Jason D Lieb

**Affiliations:** Department of Biology and Carolina Center for Genome Sciences, University of North Carolina at Chapel Hill, Chapel Hill, North Carolina, United States of America; Yale University, United States of America

## Abstract

The packaging of DNA into nucleosomes influences the accessibility of underlying regulatory information. Nucleosome occupancy and positioning are best characterized in the budding yeast *Saccharomyces cerevisiae,* albeit in asynchronous cell populations or on individual promoters such as *PHO5* and *GAL1–10*. Using FAIRE (formaldehyde-assisted isolation of regulatory elements) and whole-genome microarrays, we examined changes in nucleosome occupancy throughout the mitotic cell cycle in synchronized populations of S. cerevisiae. Perhaps surprisingly, nucleosome occupancy did not exhibit large, global variation between cell cycle phases. However, nucleosome occupancy at the promoters of cell cycle–regulated genes was reduced specifically at the cell cycle phase in which that gene exhibited peak expression, with the notable exception of S-phase genes. We present data that establish FAIRE as a high-throughput method for assaying nucleosome occupancy. For the first time in any system, nucleosome occupancy was mapped genome-wide throughout the cell cycle. Fluctuation of nucleosome occupancy at promoters of most cell cycle–regulated genes provides independent evidence that periodic expression of these genes is controlled mainly at the level of transcription. The promoters of G_2_/M genes are distinguished from other cell cycle promoters by an unusually low baseline nucleosome occupancy throughout the cell cycle. This observation, coupled with the maintenance throughout the cell cycle of the stereotypic nucleosome occupancy states between coding and non-coding loci, suggests that the largest component of variation in nucleosome occupancy is “hard wired,” perhaps at the level of DNA sequence.

## Synopsis

Every cell contains a complete copy of the genome, which is comprised of DNA. To fit the genome into the cell nucleus, DNA strands are wound around spools made of proteins called histones. By packing DNA, histones also control access to DNA, which influences when genes can be turned on and off. The authors present two main findings. First, they demonstrate a new, simple method to determine how tightly genes are wrapped around histones. Second, they use the new method to determine what happens to DNA packaging at every gene through one cycle of cell growth and division. They find that as genes get turned on throughout the cell cycle, the packaging of DNA that controls those genes becomes loose, and as genes get turned off, it becomes tight again. However, some cell cycle–regulated genes do not fit this pattern, suggesting another means of regulation for these genes. Interestingly, cell cycle–related differences in packaging at single genes are smaller than differences in packaging that are always apparent between different groups of genes. This suggests that most differences in how DNA is packaged may be controlled by the sequence of DNA itself, with smaller contributions from other factors.

## Introduction

In eukaryotic cells, DNA associates with histones to form chromatin. The fundamental unit of chromatin is the nucleosome, comprised of approximately 146 base pairs (bp) of DNA wrapped around a histone octamer [1]. In addition to packaging the DNA so that it can fit into the cell's nucleus, chromatin provides a mechanism for regulating gene expression by controlling access to the DNA template. In Saccharomyces cerevisiae (hereafter referred to as “yeast”), the mechanisms by which chromatin regulates gene expression have been investigated most thoroughly at the *PHO5* [2–18] and *GAL1–10* promoters [17,19]. Recent studies at the *PHO5* promoter demonstrate that upon transcriptional activation, promoter nucleosomes are removed by disassembly [13,14] and reassembled using histones from a source in *trans* [18].

In addition to these locus-specific studies, several groups have mapped nucleosome occupancy and positioning on a genomic scale in yeast [20–24]. Nucleosome occupancy appears to be heterogeneous throughout the genome, with regions upstream of genes being depleted of nucleosomes relative to open reading frames (ORFs) [20]. Higher-resolution data over large contiguous regions of yeast DNA revealed that approximately 70% of the nucleosomes were well positioned [23,25], and also suggested that the previously observed depletion of nucleosomes at promoters could be explained by an approximately 150-bp “nucleosome-free” region upstream of most yeast genes [23,24]. Strong evidence exists for nucleosomes regulating the accessibility of potential transcription factor binding sites [22,23,26–28] (X. Liu, C. K. Lee, J. A. Granek, N. D. Clarke, and J. D. Lieb, unpublished data).

Although the studies described above were critical for establishing the general landscape of nucleosome occupancy, they were all performed on cell populations that were not synchronized with respect to the cell cycle [20–24]. In eukaryotes, the properties of chromatin are known to vary with cell cycle phase, particularly in S-phase during DNA replication, and in mitosis as chromosomes condense. Therefore, the heterogeneity in nucleosome occupancy observed in experiments to date could represent a chromatin configuration that remains constant throughout the cell cycle, or one that is an average of distinct chromatin configurations that occur at specific cell cycle phases. Furthermore, orchestrating the expression levels of the approximately 800 cell cycle–regulated genes in yeast requires precise control of both post-transcriptional and transcriptional mechanisms of regulation, one of which is likely to be the modulation of chromatin structure at gene promoters [29–31]. Here, we used a method termed FAIRE (formaldehyde-assisted isolation of regulatory elements) [32], coupled with whole-genome DNA microarrays, to examine the state of chromatin throughout the yeast mitotic cell cycle.

## Results

### FAIRE Positively Selects for Nucleosome-Depleted Genomic Regions

Following phenol-chloroform extraction of formaldehyde-crosslinked yeast chromatin, non-coding regions of the genome are preferentially segregated into the aqueous phase, a procedure we now call FAIRE [32]. It had been previously hypothesized that this segregation occurred because covalently crosslinked protein–DNA complexes were retained at the interphase of the organic and aqueous solvents, whereas DNA that was not crosslinked (or trapped by crosslinks) escaped into the aqueous phase [32,33]. The enrichment of regulatory regions in the aqueous phase was therefore interpreted to indicate relatively inefficient crosslinking between proteins and DNA at these regions. It had been further hypothesized that FAIRE reflected heterogeneity in the distribution of nucleosomes, or differential crosslinking of modified histone tail lysines to the histone octamer, neighboring octamers, or DNA [32].

At the time FAIRE was first observed, these hypotheses were supported primarily by three observations. First, histones are by far the most abundant and readily crosslinkable protein component of chromatin and thus were likely to dominate the crosslinking profile [33–35]. Second, it was known that nucleosomes are disrupted, and in some cases lost all together, at promoters upon gene activation [10,11]. Third, in concordance with nucleosome loss at regulatory regions, promoters upstream of highly transcribed genes were more efficiently segregated to the aqueous phase in FAIRE than promoters upstream of poorly transcribed genes [32].

**Figure 1:**
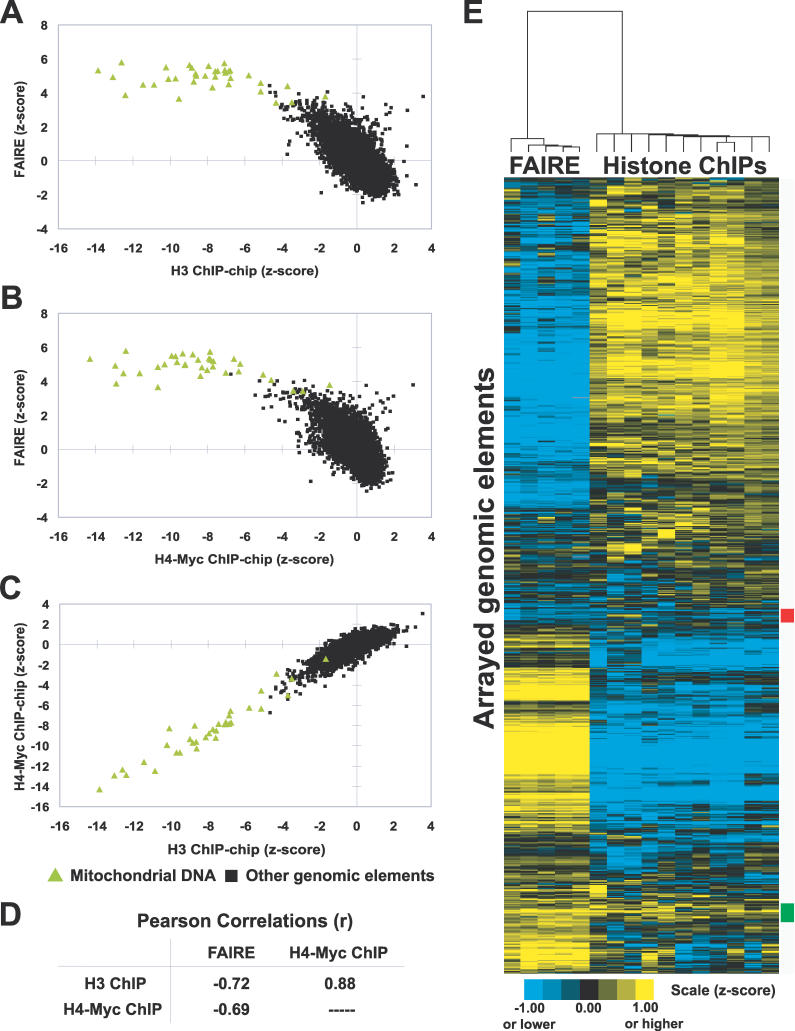
Inverse Correlation of FAIRE and Histone H3 and H4-myc ChIP-chip Data (A–C) Enrichment values from the indicated experiments were plotted. Green triangles indicate mitochondrial DNA probes, whereas black squares represent all other arrayed genomic segments. For FAIRE, data are derived from five biological replicates. The histone ChIP-chip data were previously published [20]. Individual arrays from FAIRE experiments and the histone ChIP-chips were transformed into *z*-scores, which controls for differences in variance between arrays by adjusting the standard deviation of each array to a value of 1. (D) Table of Pearson correlations between FAIRE and histone H3 and H4-myc ChIP-chip data. The significance of the correlations between FAIRE and histone ChIP-chip experiments was tested by randomly permuting the FAIRE data 1,000,000 times. A greater anti-correlation than was observed with unpermuted data was not achieved (empirical *p* < 1 × 10^−6^). (E) Hierarchical clustering by gene and by array was performed using the program Cluster [57]. All arrayed genomic elements were included. The 192 loci marked by the red box exhibit relative depletion in both FAIRE and histone ChIP-chip, but revealed no striking biases in terms of size, GC content, genomic location, or locus type (e.g., ORF, promoter, or telomeric). The 266 loci marked by the green box exhibit relative enrichment in both assays. They are mostly intergenic (76%), and of the ORFs present, 27.6% were dubious (versus 11.4% genome-wide). No other distinguishing features among the red or green groups could be identified. For all figures in this paper, although the scales are discontinuous, the data shown are continuous.

Before using FAIRE to investigate chromatin throughout the cell cycle, we sought to establish a firm relationship among FAIRE, nucleosome occupancy, and histone modification status. We performed five independent FAIRE experiments on the wild-type yeast strain BY4741. Each sample was comparatively hybridized to a DNA microarray against reference genomic DNA from BY4741. Data from these biological replicates were then used in a comparison of FAIRE to published histone H3 and H4-Myc chromatin immunoprecipitation (ChIP)-chip data [20], which were derived from samples amplified and labeled in the same manner as the FAIRE samples (Material and Methods). The correlation between FAIRE and histone ChIP-chip data is strongly negative (Figure 1A–1D). Mitochondrial DNA, which is known to be nucleosome free, was the most highly enriched class of DNA in FAIRE and the least enriched in the histone ChIP-chips. Nearly all elements depleted in the H3 and H4-Myc chips were enriched in FAIRE, and nearly all elements enriched in the histone ChIPs were depleted in FAIRE (Figure 1E). Therefore, FAIRE is a highly reproducible procedure that enriches for relatively nucleosome-free regions of the genome.

### FAIRE Resolves Nucleosome-Free Regions but Not Single Nucleosomes Using Higher-Resolution Microarrays

**Figure 2:**
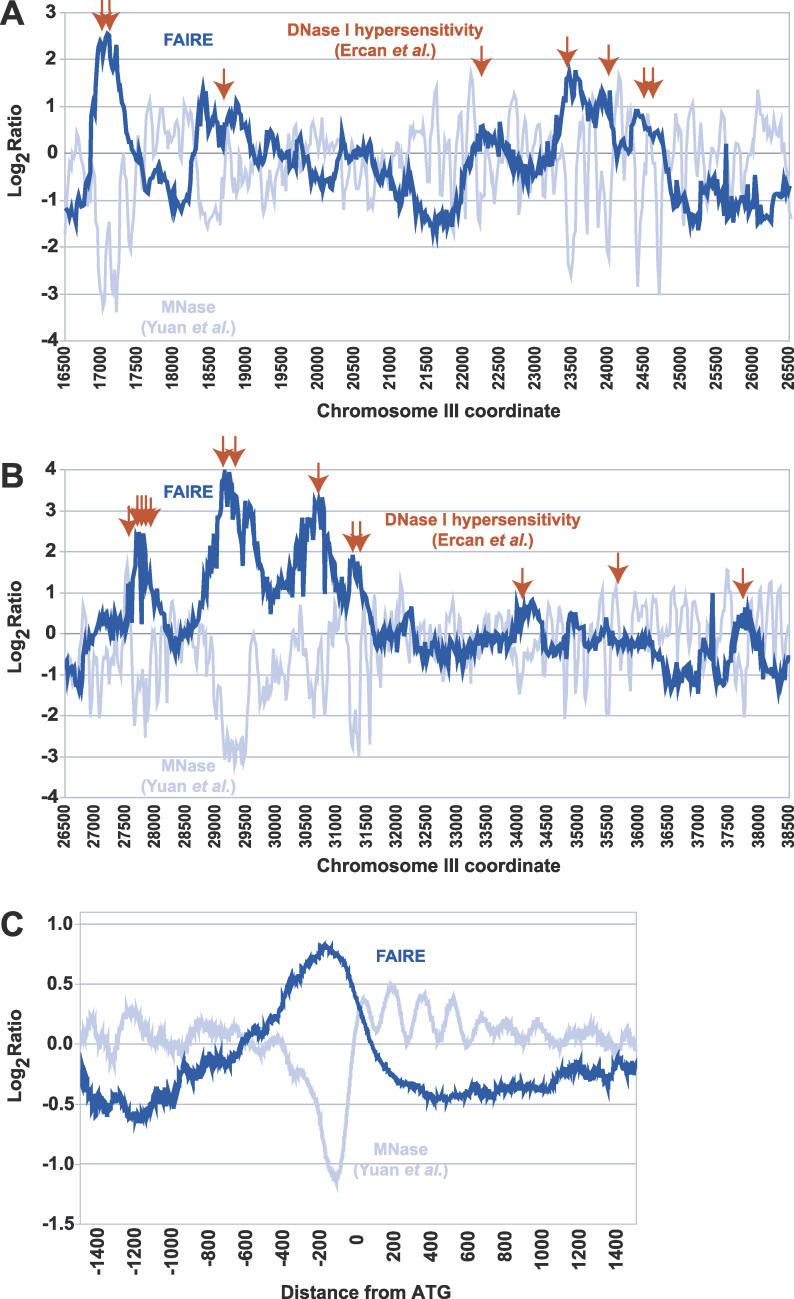
Peaks of FAIRE Enrichment Are Coincident with DNase I Hypersensitive Sites and NFRs (A) Raw data obtained through FAIRE (dark blue) and MNase treatment (light blue) [23] obtained from high-resolution tiling arrays are shown for a 10-kb region of Chromosome III (16500–26500). Red arrows represent previously mapped DNase I hypersensitivity sites [25]. (B) Same as (A), but for a 12-kb region on Chromosome III (26500–38500). (C) Data from tiling arrays were aligned by distance from start codon and averaged over 50-bp windows (step size = 1).

We tested FAIRE's ability to detect individual features related to chromatin structure, namely deoxyribonuclease I (DNase I) hypersensitive sites [25], nucleosome-free regions (NFRs) [23], and the positions of individual nucleosomes [23]. We hybridized FAIRE samples to microarrays covering 482 kilobases (kb) at 20-bp resolution [23]. First, we compared the FAIRE enrichment to the distribution of DNAse I hypersensitive sites across 45 kb of Chromosome III. Of the 34 known DNase I hypersensitive sites specific to this region [25], 28 coincided with strong peaks of FAIRE enrichment (Figure 2A and 2B). To test the relationship between NFRs and FAIRE enrichment, probes were mapped relative to the start codon of every gene represented, and FAIRE data were averaged as a function of distance from translation start sites. We found that FAIRE specifically enriches the NFRs immediately upstream of start codons (Figure 2C). However, in contrast to the micrococcal nuclease-based method employed to characterize the NFRs [23], FAIRE appears unable to resolve the individual, regularly spaced nucleosomes surrounding the NFR (Figure 2C). This resolution difference is likely due to the mechanical shearing of chromatin by sonication in FAIRE (~300 to 1,000 bp on average) versus digestion to mononucleosomes in the MNase assay.

### FAIRE Is Unaffected by Mutations in Genes Required for Specific Histone Modifications

The hypothesis that FAIRE may reflect differences in histone modification status rather than nucleosome occupancy was based on the observation that formaldehyde forms covalent crosslinks between DNA and chromatin proteins primarily through lysine residues [36]. Since many lysines in each nucleosome are capable of being modified, and modifications alter the reactivity of the lysine, it is plausible that differential histone modification could create a diversity of formaldehyde reactivity across the chromatin landscape. Each region's unique formaldehyde reactivity, dependent on the combination of histone modifications, would then be captured by FAIRE.

**Figure 3:**
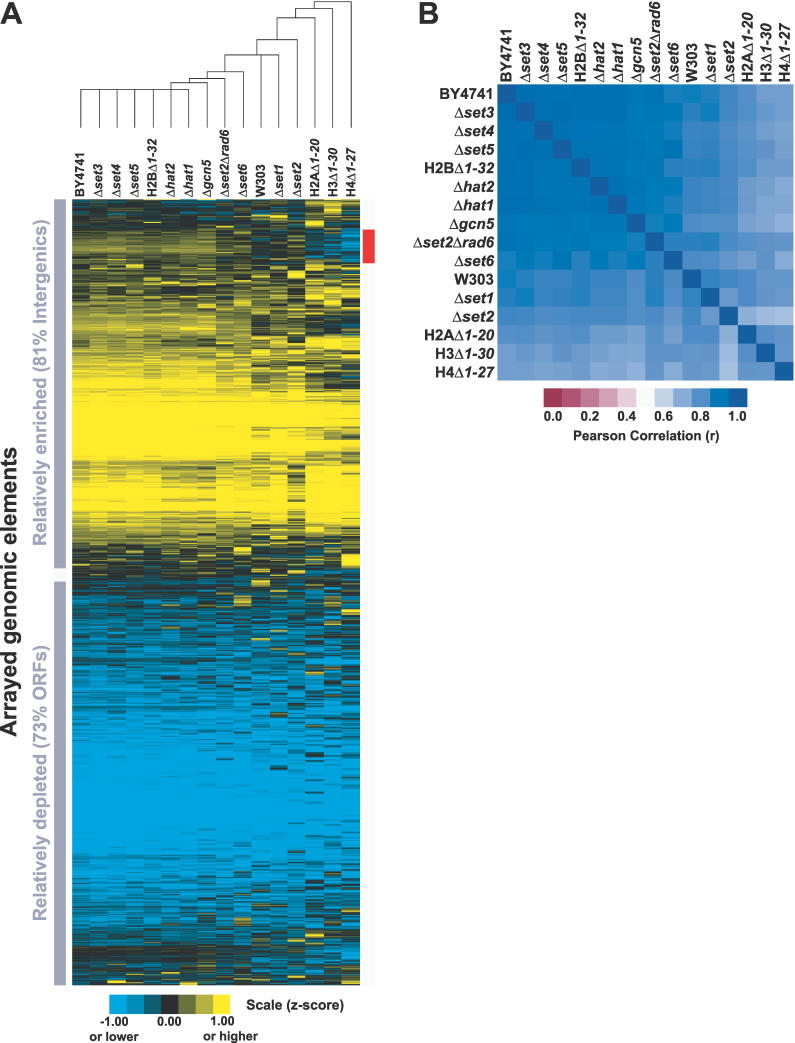
FAIRE Is Unaffected by Mutations in Genes Required for Specific Histone Modifications (A) Data are from FAIRE performed on 15 mutant strains and two wild-type strains. For each strain, normalized median log_2_ ratios from individual arrays were *z*-score transformed and averaged with biological replicates (Material and Methods). Hierarchical clustering by gene and by array was performed on all arrayed elements using Cluster [57]. The red bar indicates the 493 loci that segregate most differently in the histone tail deletion mutants. Nearly all of them (459/493, 93%) are intergenic regions. Of the 34 ORFs that differentially segregate, 12 are annotated as “dubious.” Analysis revealed no striking difference between the 493 loci and the rest of the genome with respect to GC content, and these loci are not enriched for subtelomeric or telomeric fragments. The expression level and rate of transcription of genes downstream of the intergenic loci were slightly lower than the genomic average. (B) The Pearson correlations of the FAIRE data from the 17 different mutant and wild-type strains.

To test this hypothesis, ten strains harboring deletions for histone modification enzymes and four histone tail deletion strains were subjected to FAIRE: H2AΔ*1–20,* H2BΔ*1–32,* H3Δ*1–30,* H4Δ*1–27, Δgcn5, Δhat1, Δhat2, Δset1, Δset2, Δset3, Δset4, Δset5, Δset6,* and a Δ*set2Δrad6* double knockout strain (Table 1). FAIRE was performed on each of the 14 mutant strains in duplicate, whereas wild-type strain BY4741 is represented by five biological replicates and wild-type strain W303 by a single experiment (Figure 3). Knockouts of the principal histone methyl- and acetyltransferases and histone deacetylases were of interest because acetylation, dimethylation, and trimethylation are predicted to abolish formaldehyde's reactivity with lysine. The data reveal that the histone modifications mediated by each of these enzymes are not required for fractionation by FAIRE, since each of the 14 mutant strains has a FAIRE profile very similar to the parental wild-type strain BY4741 (Figure 3). Furthermore, chromatin from a Δ*rpd3Δhda1Δhos1Δhos2Δhos3* quintuple knockout strain also showed a normal FAIRE fractionation pattern (unpublished data).

**Table 1:**
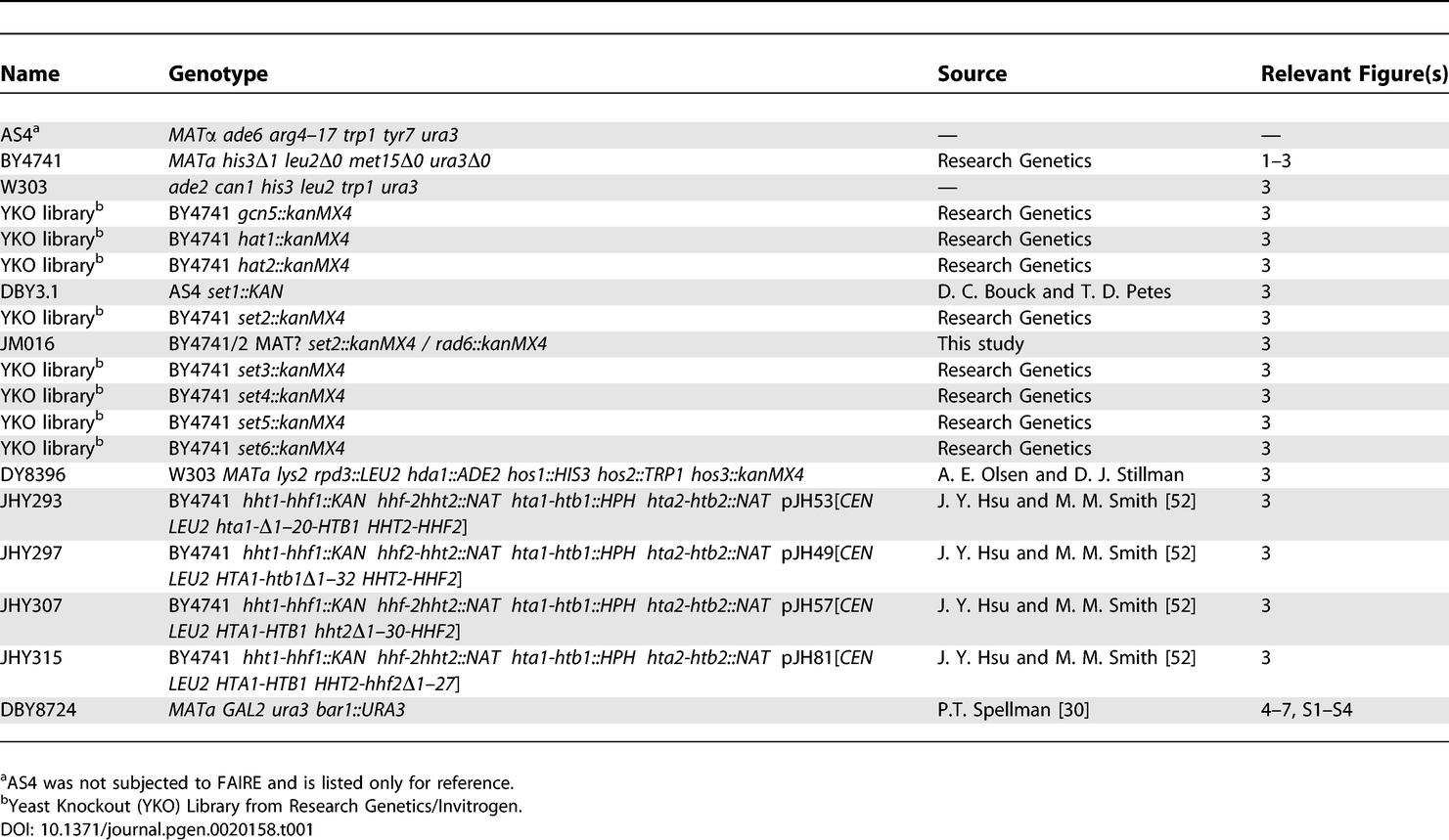
Saccharomyces cerevisiae Strains Used in This Study

The amino-terminal tails of histones H2A, H2B, H3, and H4 are the substrate for most known post-translational modifications. If histone modification status were the primary biological phenomena measured in FAIRE, a dramatic change in the fractionation would be expected in strains harboring histone tail deletions. However, H4Δ*1–27,* which is least correlated with rest of the yeast strains tested, still has a very high correlation coefficient of 0.79 to all other samples.

We conclude that the fractionation between coding and intergenic regions was essentially unaltered in all strains tested (Figure 3). The fractionation pattern of chromatin mediated by FAIRE persists in every histone modification mutant we have tested, lending support to the hypothesis that nucleosome occupancy, and not histone modification status per se, is the primary determinant of FAIRE.

### The Global Pattern of Nucleosome Occupancy Is Maintained throughout the Cell Cycle

**Figure 4:**
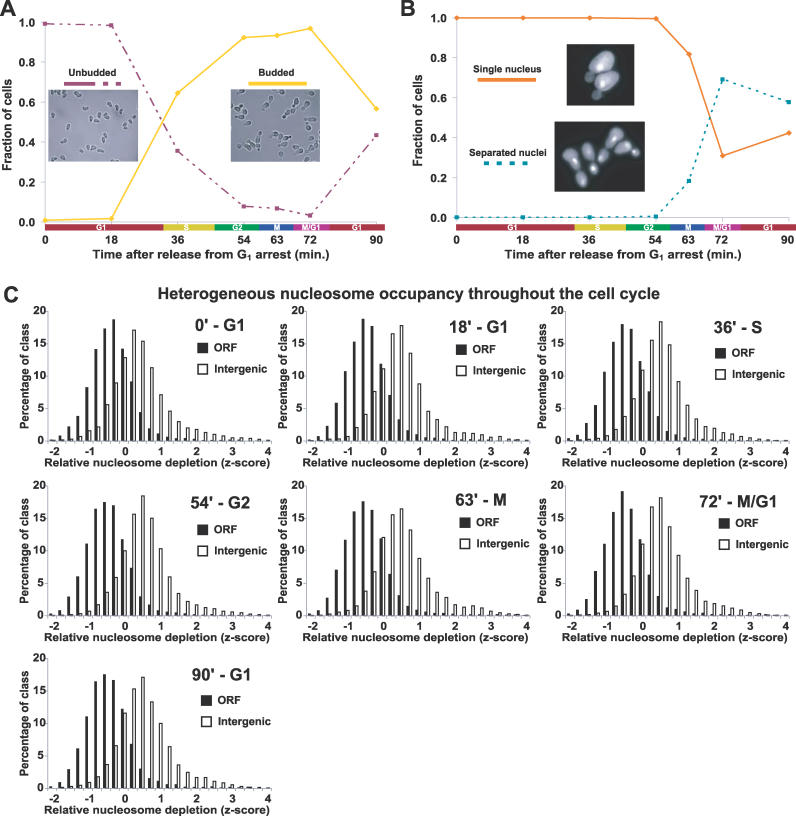
The Global Pattern of Nucleosome Occupancy Is Maintained throughout the Cell Cycle (A) To validate synchrony of cell cycle arrest and release, cells were scored as unbudded (G_1_) or budded (other phase) using brightfield images of every time point from each time course as described (Material and Methods). Each image represents one of the plotted groups, as indicated. Although the data shown were derived from a single time course, all time courses exhibit similar synchrony and growth. For a representative time course, Northern blots were performed using probes to *CLN2* and the *HHT* transcripts, which also confirmed cell cycle synchrony and progression (unpublished data). (B) Same as (A), except cells from each time point were stained with DAPI. Each yeast cell was scored as having a single nucleus (pre-mitotic) or separated nuclei (post-mitotic). Greater than 90% arrest in late G_1_ at the time of release was achieved for all cultures. Note that although the cells from the first time point (time 0) were arrested in G_1_, they were in a physiological state distinct from natural G_1_ as the result of induction of α-factor–responsive genes. (C) The distribution of *z*-scores for SGD-annotated ORFs and intergenic non-coding segments plotted for each cell cycle time point. Each histogram is represented by all biological replicates collected at that time point. The approximate cell cycle phase is given next to the time after release from G_1_ arrest. For our analysis, normalized median log_2_ ratios from each array were transformed into *z*-scores to control for differences in variance between arrays, followed by calculation of mean *z*-scores for all biological replicates (Material and Methods).

Because previous genome-scale experiments measuring nucleosome occupancy were performed on asynchronous cell cultures, it remained formally possible that variation in nucleosome occupancy was restricted to a single cell-cycle phase or a subset of the phases. We arrested cells in late G_1_ using the mating pheromone α-factor, released the cells into fresh YPD, and collected synchronized cells at seven time points spanning a single cell cycle. To verify synchronized growth and determine cell cycle phases for each time point, we monitored bud formation by light microscopy and DNA content with DAPI staining (Figure 4A and 4B). The collected time-point samples were then subjected to FAIRE, followed by microarray detection (Material and Methods). Five independent time-course experiments were performed, and thereby each of the seven time points is represented by at least three (and up to five) high-quality data points.

The distribution of FAIRE enrichment values for intergenic regions was compared to that of ORFs throughout the cell cycle. FAIRE was able to detect the previously observed heterogeneous nucleosome occupancy genome-wide at every time point assayed throughout the cell cycle (Figure 4C). Because all time points showed a similar global pattern of FAIRE enrichment, we conclude that the overall state of the nucleosome occupancy landscape is maintained throughout the cell cycle.

### Release from Mating Pheromone Induces Increased Nucleosome Occupancy at *FIG1, FIG2,* and *FIG3* Promoters

**Figure 5:**
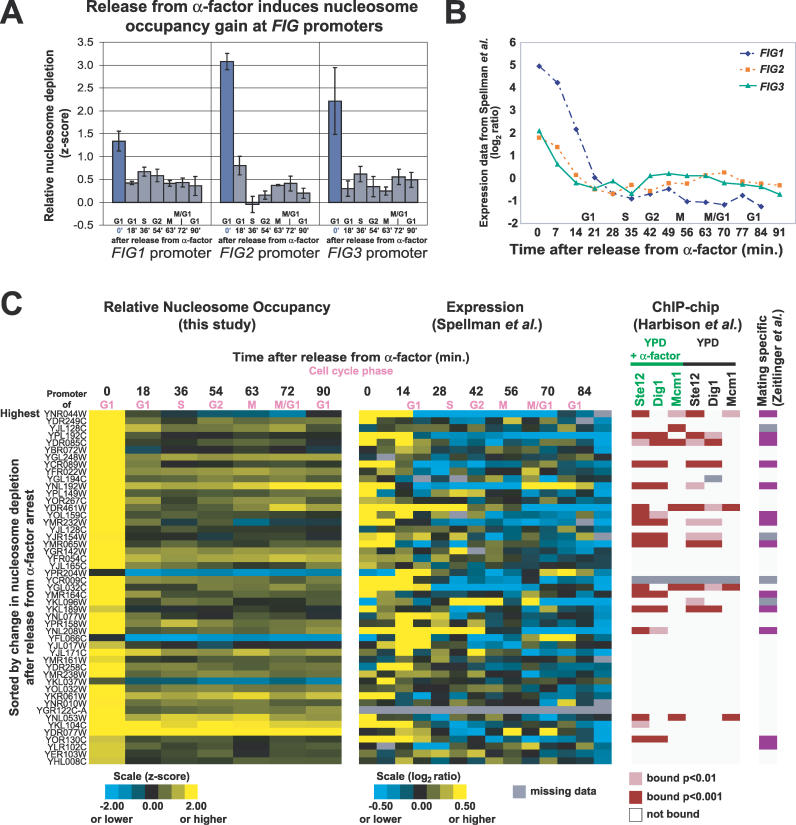
Release from Mating Pheromone Induces Increased Nucleosome Occupancy at *FIG1, FIG2,* and *FIG3* Promoters (A) Relative nucleosome depletion at the *FIG1, FIG2,* and *FIG3* promoters upon release from mating pheromone. Plotted values are *z*-scores derived from FAIRE data. At 0 min, cells are in α-factor–induced arrest. Error bars are ± SEM. (B) Previously obtained expression data for *FIG1, FIG2,* and *FIG3* after release from α-factor [30]. (C) Promoters exhibiting a large increase in nucleosome occupancy after release from α-factor are shown (FAIRE *z*-score decrease greater than 1), with corresponding gene expression data [30] and ChIP-chip data [40]. In the far-right column, a purple box indicates that a downstream gene was characterized as mating specific by Zeitlinger et al. [39].

To test the ability of our assay to detect nucleosome occupancy changes at promoters, we analyzed nucleosome occupancy at the promoters of three genes known to respond to mating pheromone. *FIG1, FIG2,* and *KAR5/FIG3* are important for the yeast mating response and are induced and highly expressed in the presence of mating pheromone [37]. Activation of *FIG1* by mating pheromone results in nucleosome occupancy loss at its promoter [38]. The FAIRE data provide evidence for the converse effect by showing that removal of the mating pheromone results in less-efficient FAIRE enrichment of the *FIG1* promoter, indicating a nucleosome occupancy gain that is sustained well after pheromone release (Figure 5A). This gain of nucleosome occupancy, which correlates with independently obtained expression data, is also characteristic of the promoters of *FIG2* and *KAR5/FIG3* (Figure 5A and 5B).

To test whether FAIRE was able to identify other genes responsive to mating pheromone, we sorted the unidirectional promoters by the difference in FAIRE enrichment before and after mating pheromone release. The top promoters (those with a *z*-score difference greater than 1) demonstrate a significant increase in nucleosome occupancy (decreased FAIRE signal) upon release from mating pheromone that correlates to lower gene expression levels [30] (Figure 5C). Additionally, the identified promoters were more likely to be bound by the transcription factors Ste12, Dig1, and Mcm1, all of which are involved in mating-pheromone response (Figure 5C) [39]. Finally, the list of putative pheromone-responsive promoters is also enriched for independently defined “mating-specific genes” [39]. Of the 45 top promoters, 13 (28.9%) are bound by transcription factors in a mating-specific fashion, versus 47/2,442 (1.9%), of other unidirectional promoters (chi-square *p*-value = 1.6 × 10^−31^) [39,40] (Figure 5C). Therefore, FAIRE-based measurements of nucleosome occupancy at gene promoters during response to a stimulus or environmental change can be used as a predictor of gene function.

### Nucleosome Occupancy at Cell Cycle–Regulated Promoters Varies with the Cell Cycle

Based on the general observation that nucleosome occupancy at a promoter is inversely proportional to the transcription rate of its downstream gene [20], we hypothesized that nucleosome occupancy at the promoters of cell cycle–regulated genes would be reduced specifically at the cell cycle phase in which that gene exhibited peak expression. To test our hypothesis, we used data from a previous study that found approximately 800 genes to be cell cycle regulated [30]. We focused primarily on genes with unidirectional (single) promoters, which constitute close to half of the 800. The cell cycle–regulated genes were previously grouped into five categories with somewhat arbitrary dividing lines: G_1_, S, S/G_2_, G_2_/M, and M/G_1_ (these categories are used in the online supplement of [30]). We used these classifications to assign each promoter to the same cell cycle group as its downstream gene. For our initial analysis, we focused on changes in nucleosome occupancy within each promoter as the cells progressed through the cell cycle. To do this, we centered the data for each promoter by the median of the *z*-scores recorded at all seven time points, and used these centered *z*-score values for downstream analyses (Material and Methods).

**Figure 6:**
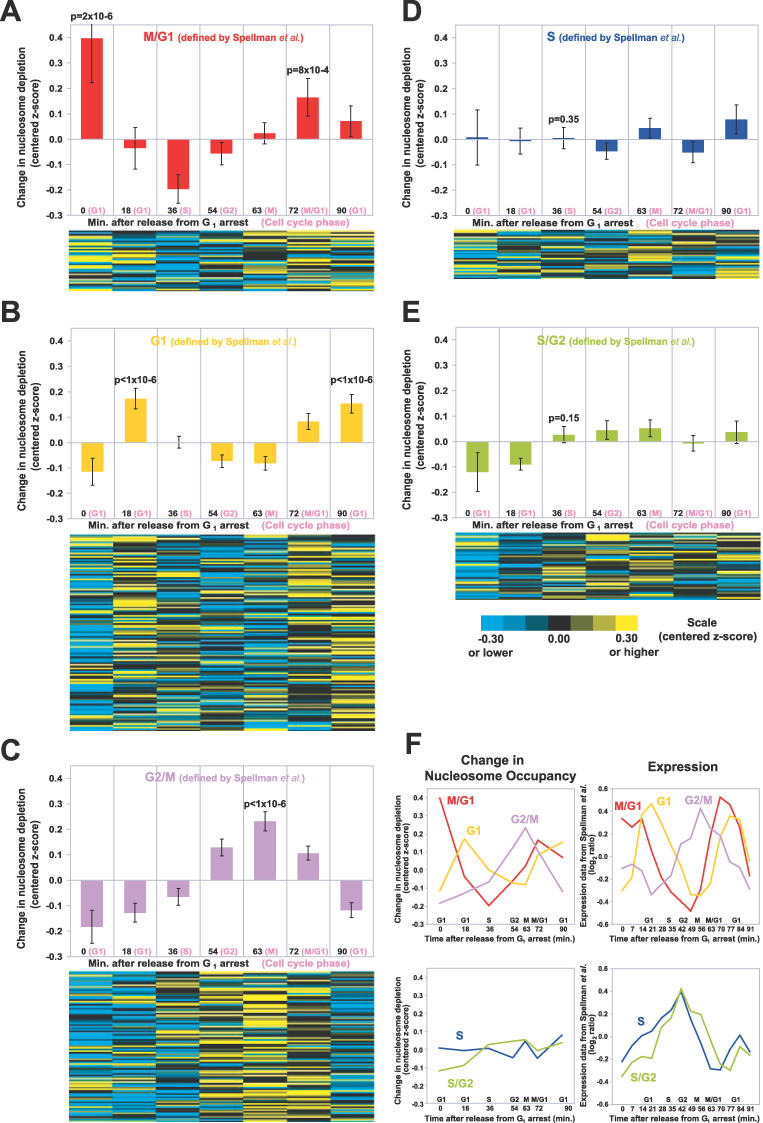
Nucleosome Occupancy at Cell Cycle–Regulated Promoters Varies with the Cell Cycle (A) In the upper panel, the mean of centered *z*-scores were plotted for the promoters of M/G_1_ genes (as defined by [30]). Only the unidirectional promoters of cell cycle–regulated genes were analyzed. The error bars are ± SEM. The *p*-values for peaks were derived from 1,000,000 permutations on data from the unidirectional promoters (Material and Methods). In the lower panel, data from the individual promoters are shown. The promoters are ordered according to the timing of their expression in the cell cycle [30]. (B) Same as (A), but for G_1_ promoters. (C) Same as (A), but for G_2_/M promoters. (D) Same as (A), but for S promoters. (E) Same as (A), but for S/G_2_ promoters. (F) The average chromatin profiles derived from FAIRE data (left), plotted alongside the average expression profiles from Spellman et al. (right) [30].

We found a positive correlation between nucleosome depletion at cell cycle–regulated gene promoters and the periodic expression of the cell cycle–regulated genes. In particular, the G_1_, G_2_/M, and M/G_1_ promoters demonstrated periodicity according to the cell cycle (Figure 6A–6C). The bidirectional promoters of cell cycle–regulated genes, which represent a completely independent sample, showed similar results despite the possible competing influence from a second gene (Figure S1).

### Nucleosome Occupancy at S and S/G_2_ Promoters Does Not Correlate with the Cell Cycle

The nucleosome occupancies of S and S/G_2_ promoters did not correlate with the timing of the cell cycle (Figure 6D and 6E). The distinct behavior of the S and S/G_2_ promoters may reflect properties of the deposition of new nucleosomes during DNA replication (Discussion). It is noteworthy that S/G_2_ genes were originally distinguished from “S” genes only by their slower decline in relative mRNA levels after this common peak [30].

### The Nucleosome Occupancy of Most, but Not All, G_1_, G_2_/M, and M/G_1_ Promoters Fluctuate Strongly with Cell Cycle Phase

**Figure 7:**
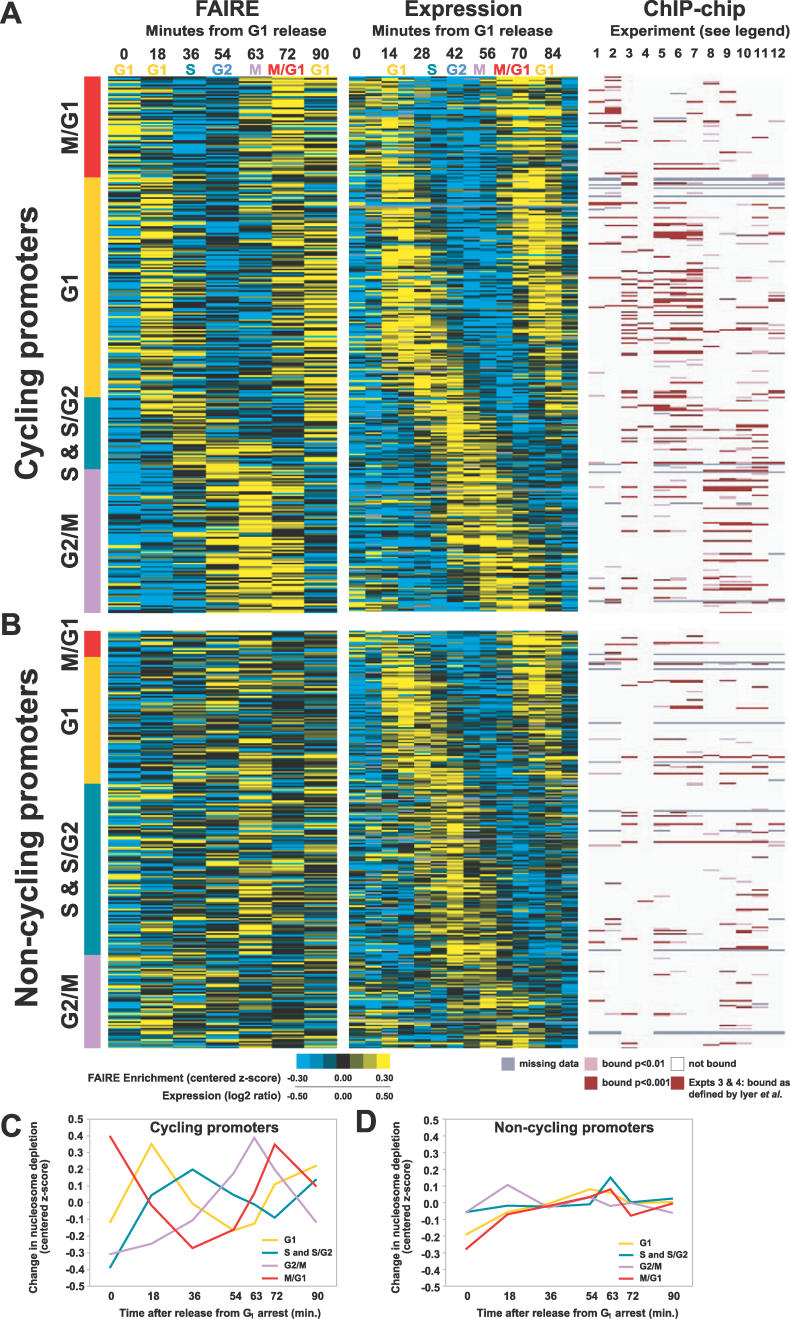
Classification of Cell Cycle–Regulated Promoters For panels (A) and (B), the approximate cell cycle stage corresponding to each time point in the FAIRE and expression [30] time course is listed along the top. ChIP-chip experiments are numbered 1 through 12 as follows (1) Ace2, (2) Swi5, (3) SBF, (4) MBF, (5) Swi4, (6) Swi6, (7) Mbp1, (8) Mcm1, (9) Ndd1, (10) Fkh2, (11) Fkh1, and (12) Stb1. For experiments 3 and 4 [42], a red bar indicates binding as defined by the authors. For experiments 1, 2, and 5–12 [40], a dark bar indicates binding at *p* < 0.001, a lighter bar indicates binding at *p* < 0.01. White indicates a lack of binding, and missing data are indicated by gray. Each cell cycle promoter was classified into the “cycling” or “non-cycling” group based on visual inspection of its nucleosome occupancy profile. All loci in this figure and their classifications can be found in Table S1. (A) Promoters with FAIRE signals that fluctuated with the cell cycle (proportion cycling: G_1_, 63%; S & S/G_2_, 30%; G_2_/M, 61%; and M/G_1_, 79%). For the FAIRE experiments, yellow indicates enrichment, blue depletion. For expression, yellow indicates high relative expression, blue low. (B) Promoters with FAIRE signals that did not fluctuate with the cell cycle. (C) The average chromatin profiles derived from FAIRE data for cycling promoters and (D) non-cycling promoters.

We have presented evidence that as a group, promoters of cell cycle–regulated genes expressed in G_1_, G_2_/M, and M/G_1_ exhibit changes in nucleosome occupancy that correspond to cell cycle regulation (Figure 6F). Inspection of the individual promoters of the cell cycle–regulated genes within each of these groups revealed that most, but not all, promoters demonstrated such fluctuations (G_1_, 63%; G_2_/M, 61%; and M/G_1_, 79%) (Figure 7A–7D, Table S1, Figure S2). Promoters that experienced cell cycle–related fluctuations in nucleosome occupancy (hereafter referred to as “cycling”) were more likely to be bound by known cell cycle transcription factors than those that did not experience nucleosome fluctuations (Figure 7A and 7B). SBF (comprised of Swi6 and Swi4) and MBF (comprised of Swi6 and Mbp1) are heterodimeric transcription factor complexes important for the expression of G_1_-specific genes [41]. Of the 135 G_1_ cycling promoters, 54 are bound by SBF or MBF, whereas only 13 of the 78 that did not exhibit fluctuations were bound by either (chi-square *p*-value = 4.1 × 10^−4^) [40,42]. In addition, cycling G_2_/M gene promoters were more likely to be bound by Mcm1, Fkh2, and Ndd1, which are known to be important for the expression of G_2_/M genes [41]. Of 85 G_2_/M cycling promoters with ChIP data, 16 bind all three transcription factors (at *p* < 0.01), whereas none of the 55 non-cycling promoters with ChIP data bind all three (chi-square *p*-value = 6.3 × 10^−4^) (Figure 7A and B) [40]. Furthermore, the previously defined “cycling score” [30] is higher for genes at which the nucleosome occupancy of the promoter also cycles (unpublished data). Therefore, genes likely to be regulated at the level of transcription can be identified by the characteristic nucleosome occupancy profiles of their promoters. We also note that the promoters of major cell cycle regulators themselves (*CLB1, CLB2, CLB5, CLB6, CLN1, CLN2, CLN3, CDC20, PCL2, PCL9, SIC1,* and *SWE1*) exhibited cell cycle–dependent changes in nucleosome occupancy (Figure S3).

### G_2_/M Promoters Are Relatively Depleted of Nucleosomes throughout the Cell Cycle

**Figure 8:**
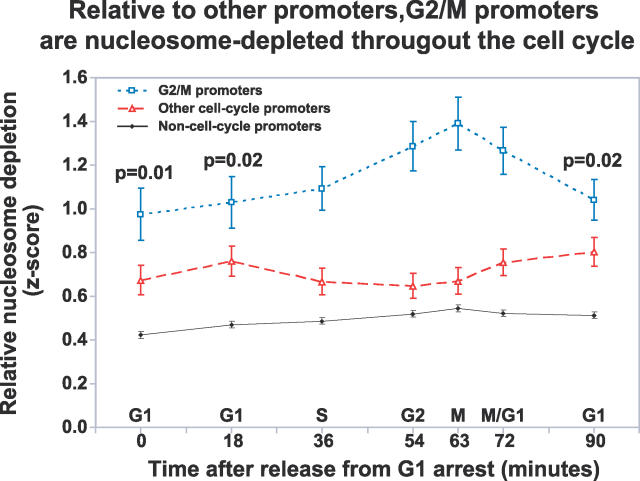
Relative to Other Promoters, G2/M Promoters Are Nucleosome Depleted throughout the Cell Cycle. The broken blue line indicates the mean *z*-score of FAIRE enrichment of unidirectional G_2_/M promoters at each time point throughout the cell-cycle. The mean of all other cell cycle unidirectional promoters (broken red line) and non–cell cycle promoters (solid black line) are also shown. The error bars indicate ± SEM. The *p*-values for the significance of the difference between the blue and red lines were derived from 1,000,000 permutations of the data (Material and Methods). The *p*-values for 0, 18, and 90 are relevant since they represent “baseline” occupancy (time points comprising the peak were all less than 0.0002.)

By examining general nucleosome occupancy within individual cell cycle groups, we found that the unidirectional promoters of G_2_/M genes tend to be more depleted of nucleosomes at all time points throughout the cell cycle relative to other unidirectional promoters of cell cycle–regulated genes and all other unidirectional promoters, irrespective of periodic transcription or nucleosome occupancy changes (Figure 8). This difference in the baseline nucleosome occupancy may indicate a specialized promoter organization required for the expression of genes during mitotic chromosome condensation or during mitosis [43] (see Discussion).

### Among the Promoters of Cell Cycle–Regulated Genes, Those That Respond to *CLN3* and *CLB2* Induction Display Particularly Large Changes in Nucleosome Occupancy

Cell cycle progression is tightly regulated in yeast, and the core elements of this regulation are highly conserved throughout eukaryotic lineages. The cell cycle is regulated in part by various cyclins that interact with a cyclin-dependent kinase (CDK) to regulate its activity. In *S. cerevisiae, CLN3* and *CLB2* encode cyclins that activate Cdc28, the main CDK, to promote cell cycle transitions. Cln3 is involved in the G_1_ to S transition, and Clb2 is involved in the G2 to mitosis transition. More than half of the cell cycle–regulated genes respond to artificial induction of either *GAL-CLN3* or *GAL-CLB2,* indicating that *CLN3* and *CLB2* are major controllers of cell cycle–regulated gene expression [30]. As expected, cell cycle–regulated genes induced by *GAL-CLN3* expression were generally G_1_ genes, whereas those responding to *GAL-CLB2* induction were generally G_2_/M genes.

**Figure 9:**
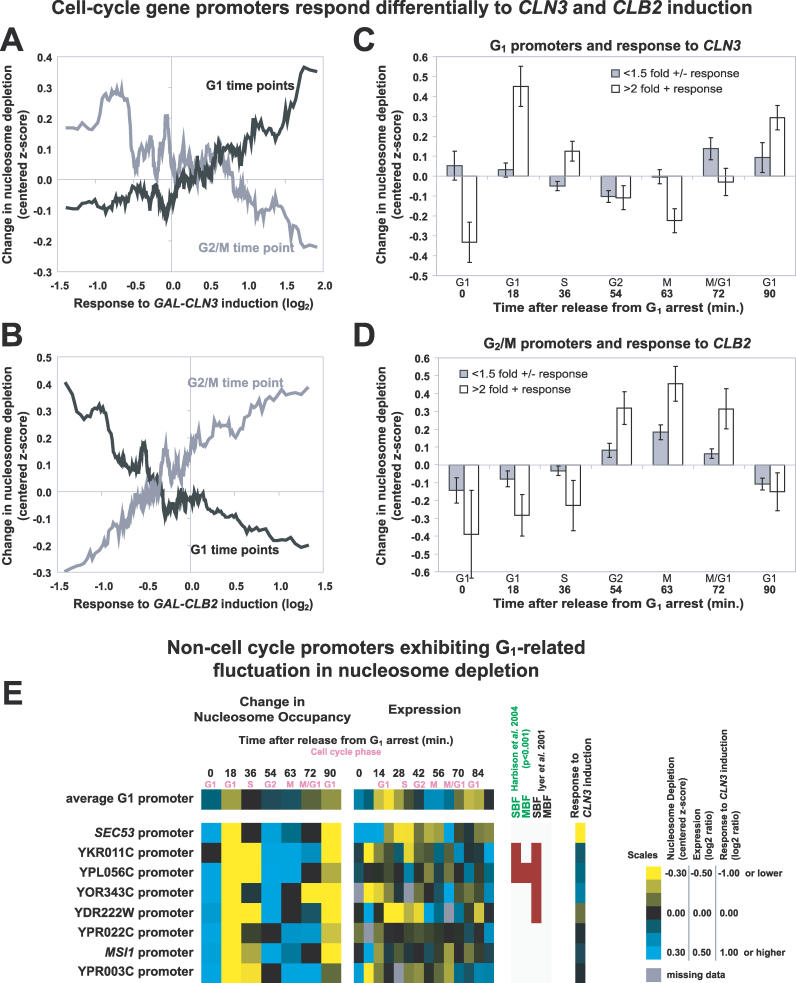
Relationship between Cyclin Induction and Changes in Nucleosome Depletion at the Promoters of Cell Cycle–Regulated Genes (A) Genes with unidirectional promoters were ordered according to their response to *CLN3* induction [30]. The moving average of the response of the corresponding genes to *CLN3* induction is plotted on the *x*-axis (window = 25, step = 1), whereas *y*-axis values represent the moving average of FAIRE enrichment (centered *z*-scores, window = 25, step 1) at the indicated time points (G_1_ in black, 18 and 90 min; G_2_/M in gray, 63 min). Therefore, positive *y*-axis values indicate nucleosome depletion. (B) Same as (A) but for *CLB2* induction. (C) Unidirectional promoters of G_1_ genes were grouped by response to *GAL-CLN3* induction (filled bars, greater than 2-fold change; open bars, less than 1.5-fold). The mean of the centered *z*-scores was plotted. The error bars are ± SEM. (D) Same as (C), but for G_2_/M gene promoters grouped by response to *GAL-CLB2* induction. (E) Examples of promoters upstream of genes not previously annotated as cell cycle–regulated that exhibited G_1_-related fluctuation in nucleosome depletion are shown alongside corresponding gene expression data [30] and ChIP-chip data for G_1_ transcription factors [40,42].

We examined the nucleosome occupancy of promoters of cell cycle–regulated genes induced by *GAL-CLN3* [30]. These promoters were nucleosome depleted during G_1_ time points (18 min and 90 min) and most nucleosomal at G_2_/M (63 min), whereas the promoters of genes repressed by *GAL-CLN3* expression [30] were most nucleosomal at G_1_ and depleted of nucleosomes at G_2_/M (Figure 9A). The inverse relationships were observed for the promoters of genes affected by *GAL-CLB2* expression (Figure 9B). Induced promoters were nucleosome depleted during G_2_/M (63 min) and most nucleosomal at G_1_ time points (18 min and 90 min), whereas the promoters of genes repressed by *GAL-CLN3* expression [30] were most nucleosomal at G_2_/M and depleted of nucleosomes at G_1_.

We next compared the nucleosome occupancy changes at the promoters of cell cycle–regulated genes that were positively induced by *GAL-CLN3* or *GAL-CLB2* to cell cycle–regulated genes that were not strongly induced by *GAL-CLN3* or *GAL-CLB2*. Comparisons were made within the respective cell cycle phase groups (G_1_ for *GAL-CLN3* and G_2_/M for *GAL-CLB2*). We defined “induced” genes as those increasing more than 2-fold and genes “not induced” as those changing less than 1.5-fold in either direction. The promoters of G_1_ genes induced by *GAL-CLN3* were more nucleosome depleted during G_1_ and also showed greater amplitude in periodicity through the cell cycle compared to the promoters of G_1_ genes not induced by *GAL-CLN3* (Figure 9C). We used a two-sample Student's *t*-test to find that the means of the groups were significantly different during G_1_ time points (*p* = 2.9 × 10^−4^ for time 18, *p* = 0.045 for time 90). Examination of the G_2_/M gene promoters at the 63-min time point yielded analogous results (*p* = 0.020 for time 63) (Figure 9D).

A plausible explanation for the greater nucleosome fluctuations observed for *CLN3*- or *CLB2*-responsive genes was illuminated by re-examination of the cell cycle expression data. The RNA levels of genes that respond to *CLN3* or *CLB2* induction experience greater changes in relative abundance throughout the natural cell cycle than those that do not respond to *CLN3* or *CLB2* induction (Figure S4). The greater nucleosome fluctuations observed for promoters of *CLN3*- or *CLB2*-responsive genes suggest more transcription initiation events at the time of peak RNA levels, rather than regulation by a post-transcriptional mechanism. Further support for this hypothesis was obtained by analysis of the data for all unidirectional promoters of cell cycle–regulated genes, which revealed a positive correlation between the amplitude of nucleosome occupancy and the amplitude of RNA level throughout the time course (Figure S4). The greater nucleosome depletion at these promoters would then be explained by the known positive relationship between transcriptional initiation and nucleosome loss [20,32].

### Cell Cycle–Correlated Nucleosome Depletion at Promoters of Genes Not Annotated as Cell Cycle Regulated

We probed the dataset for promoters of genes that were not annotated as being cell cycle regulated but exhibited cell cycle–related fluctuations in nucleosome occupancy (Figure 9E). We limited our focus to unidirectional promoters that exhibited G_1_-related fluctuations. Limiting the search to G_1_-related fluctuations ensured greater statistical significance because the time course captured two separate G_1_ phases. Data from individual unidirectional promoters were tested for correlation to the average G_1_ FAIRE profile (*r* > 0.6) and for a minimum change in FAIRE enrichment (*z*-score change > 1.0) over the entire time course.

Of the 42 promoters that met these criteria, several are upstream of genes for which, upon closer inspection, there exists independent evidence of cell cycle regulation. For example, *SEC53* is highly induced by *CLN3* [30] whereas the promoters of YKR011C, YPL056C, YOR343C, and YDR222W are bound by G_1_ transcription factors [40,42] (Figure 9E). Overall, five of the 42 (11.9%) are bound by G_1_ transcription factors, demonstrating an enrichment over the rest of the unidirectional promoters tested (48/2,486, or 1.9%, chi-square *p*-value = 7.7 × 10^−6^) [42].

Most of the identified promoters, however, do not yet have reported connections to the cell cycle. For example, the promoters of YPR022C, *MSI1,* and YPR003C exhibit strong cell cycle–related changes in FAIRE enrichment but are not annotated as being cell cycle regulated, based on fluctuations in RNA levels (Figure 9E). RNA transcripts from these genes may be subject to post-transcriptional regulation that would prevent their detection, such as rapid RNA degradation. Cell cycle–regulated transcriptional initiation, in combination with a post-transcriptional regulatory mechanism, could be a means for allowing cell cycle expression of genes under specific environmental or developmental conditions.

## Discussion

### Measurement of Nucleosome Occupancy throughout the Cell Cycle

The creation of maps of nucleosome occupancy throughout the cell cycle presented several challenges regarding experimental design and data analysis. These included (1) establishing a robust method for the measurement of nucleosome occupancy, (2) ensuring a high degree of cell cycle synchrony, (3) sampling the cell cycle at high enough temporal resolution and with enough independent replicates to capture cell cycle fluctuations in nucleosome occupancy while maintaining a feasible experimental design and cost structure, (4) use of relatively low-resolution DNA microarrays, and (5) registration of our FAIRE dataset to gene expression measurements obtained from completely independent cell cycle experiments performed by a different group.

Despite these challenges, the results provide clear evidence that the promoters of G_1_, G_2_/M, and M/G_1_ cell cycle–regulated genes are depleted of nucleosomes at the phase during which the downstream genes are most highly expressed. The results presented here extend studies that examined the promoter of *PHO5* [2–18] and provide further evidence that the loss of nucleosome occupancy in coordination with transcriptional activation is a fundamental feature of transcriptional regulation in eukaryotes. The periodic nature of these changes was demonstrated by the G_1_ gene promoters, which exhibited strong nucleosome depletion early in the first cell cycle, returned to baseline, and then were again nucleosome depleted at the start of the second cell cycle.

However, cell cycle variation of nucleosome occupancy was not generally observed at the promoters of S and S/G_2_ genes. Although we cannot rule out the possibility that S and S/G_2_ peaks were not captured ideally by our selected time points, one plausible explanation for the lack of cell cycle–specified variation from the promoters of S and S/G_2_ genes is that the process of DNA replication causes changes in nucleosome occupancy that obscures our ability to measure transcription-associated changes.

We also failed to observe any indication of mitotic chromosome condensation in the FAIRE results. During interphase, yeast chromosomes are compacted to a degree similar to that observed in other eukaryotes, including humans [44]. However, at mitosis, human chromosomes condense 5- to 10-fold [45], whereas yeast chromosomes seem to undergo little further compaction, perhaps 2-fold [44]. In addition to the possibility that compaction in yeast is subtle, our failure to observe any systemic difference in the FAIRE data during mitosis could simply reflect the possibility that the nucleosomal level of organization assayed by FAIRE may be unaffected by the higher-order compaction that occurs during mitosis.

### A “Hard Wired” Mechanism May Account for Most Variation in Nucleosome Occupancy

We found that despite the processes of DNA replication and mitotic chromosome condensation, the most pronounced global features of the nucleosome occupancy landscape persist throughout the cell cycle. Foremost among these is the consistently higher nucleosome occupancy observed in coding regions relative to non-coding regions. This maintenance of global chromatin structure suggests that the nucleosome occupancy of most genomic loci is relatively “hard wired.” perhaps at the level of DNA sequence [22,28,46], and that the dynamic fluctuations we observe are overlaid upon this framework. The very low “baseline” level of nucleosome occupancy observed throughout the cell cycle in the unidirectional promoters of G_2_/M genes provides further support for this hypothesis. Perhaps the nucleosome paucity at most G_2_/M promoters plays a role in sustaining accessibility to regulatory information as the genome becomes more condensed in preparation for mitotic division. Consistent with this notion, the activities of SWI/SNF chromatin remodelers and the histone acetyltransferase Gcn5p are generally required for gene expression during mitosis [43].

### Nucleosome Occupancy: Cause or Effect?

Our studies do not allow one to distinguish cause and effect regarding the relationship between nucleosome occupancy, transcription factor binding, and transcriptional initiation. The situation is likely to be complex and locus dependent. However, other studies designed to address this question indicate that nucleosome occupancy can play an instructive role in transcription factor targeting [22] (X. Liu, C. K. Lee, J. A. Granek, N. D. Clarke, and J. D. Lieb, unpublished data). Therefore, regulation of nucleosome occupancy is likely to be an important factor in the cell cycle regulation of transcription.

Combinatorial control of the *HO* gene is a well-studied example of complexity in control of transcriptional activation. The *HO* gene encodes an endonuclease whose activity results in switching of the mating types between **a** and α. *HO* expression in late G_1_ is tightly regulated through stepwise recruitment of Swi5, SWI/SNF, SAGA, and SBF [47,48]. Prior to *HO* transcription, histones at the promoter are acetylated [47], and our data show an exceptional loss of nucleosome occupancy at the *HO* promoter, starting at the M/G_1_ transition and continuing into the second G_1_ phase of the time course (note that, as expected, *HO* is not expressed in the first cell cycle following release from α-factor [49]). It is likely that the nucleosome depletion we observe occurs at the *HO* promoter as a result of SWI/SNF and SAGA activities, which then facilitate the binding of SBF.

At some promoters it is clear that fluctuations in nucleosome occupancy occur even after a subset of regulatory factors are bound. For example, Fkh2 and Mcm1 form a repressor complex that continuously occupies promoters of many *CLB2*-induced genes [50]. Although binding of Fkh2 and Mcm1 is not cell cycle regulated, the binding of Ndd1 to the forkhead-associated (FHA) domain of Fkh2 is cell cycle regulated, and provides the switch that activates the previously repressive complex [50,51]. Nonetheless, our data show that nucleosome occupancy at promoters bound by these transcription factors vary with cell cycle phase. Whether the changes in nucleosome occupancy facilitate Ndd1–Fkh2 interaction or occur as a consequence of Ndd1 binding to the repressor complex is not known.

### FAIRE for Detection of Nucleosome-Depleted Genomic Regions

We have presented evidence that FAIRE specifically enriches genomic regions that are relatively depleted of nucleosomes. In addition, peaks of FAIRE enrichment were concordant with classically defined DNase I hypersensitive sites and NFRs. The current implementation of FAIRE appears to provide a resolution just lower than that of a single nucleosome. FAIRE is technically simpler than histone ChIP-chips or DNase treatment, is highly reproducible, and requires no antibodies. Therefore, FAIRE holds promise as a high-throughput, ChIP- and nuclease-independent method for assaying nucleosome occupancy and changes in nucleosome occupancy genome-wide in yeast and other organisms. In addition, FAIRE may be useful for measuring nucleosome occupancy in strains in which conventional ChIP is impractical, such as those containing TAP fusion proteins. FAIRE profiles are likely to integrate many factors that influence nucleosome positioning and stability, including changes in nucleosome composition, histone modifications, and modifications to DNA.

## Materials and Methods

### Yeast strains.

Table 1 lists all strains used in this study. DBY3.1 was provided by D. C. Bouck and T. D. Petes. It contains a complete deletion of *SET1* (from ATG to nonsense codon), made with PCR to replace the *SET1* gene with *kanMX* using AS4 as the parental strain. JM016 was created by mating Δ*rad6* (BY4741 *rad6::kanMX4*) and Δ*set2* (BY4742 *set2::kanMX4*), sporulating, and selecting for the haploid double mutants. DY8396 was provided by A. E. Olsen and D. J. Stillman. The N-terminal histone tail deletions were provided by J. Y. Hsu and M. M. Smith, and were constructed as previously described [52]. For all cell cycle experiments, the yeast strain DBY8724 *(MATa GAL2 ura3 bar1::URA3)* was used [30].

### Culture conditions for cell cycle time courses.

For each cell cycle time course, yeast were grown in YPD (1% yeast extract, 2% peptone, 2% dextrose) at 30 °C with shaking to an OD_600_ of approximately 0.2. 1 μl of α-factor (final concentration 42 ng/ml) was added and shaking at 30 °C was continued for 120 min to ensure arrest in late G_1_. After this treatment, microscopy verified that at least 90% of the cells were unbudded. The arrested culture was spun for 5 min at 650 *g*. The YPD was decanted, and the culture was resuspended in fresh YPD, releasing the cells from arrest. At this point, 50 ml of “time 0” sample was removed and fixed with formaldehyde as described below (FAIRE procedure). The remaining culture was returned to growth at 30 °C with shaking, and 50 ml of culture was removed and fixed with formaldehyde at 18, 36, 54, 63, 72, and 90 min after release from α-factor arrest. Small samples of formaldehyde-fixed cells from each time point were set aside for bud counts and DAPI staining.

### Bud counts and DAPI staining.

For bud counts, formaldehyde-fixed cells were sonicated for 3 to 5 s using a Branson Digital Sonifier (Branson, Danbury, Connecticut, United States) at 15% amplitude and then concentrated onto glass slides. Cells were observed using a Nikon TE2000 microscope (Nikon, Tokyo, Japan) with an oil-immersion lens, and brightfield images were acquired with an ORCA II ER cooled CCD camera (Hamamatsu Photonics, Hamamatsu City, Japan) and MetaMorph software (Molecular Devices, Sunnyvale, California, United States).

For DAPI (4',6-diamidino-2-phenylindole) staining, formaldehyde-fixed cells were sonicated for 3 to 5 s, spun down, washed with PBS. Cells were resuspended in water and two volumes of 70% ethanol for 20 min at room temperature. After washing again with PBS, DAPI (1 mg/ml) was added to a final concentration of 1 μl/ml. Cells were left in the dark for 10 min at room temperature, washed a final time with PBS, and concentrated on glass slides for analysis. Epifluorescence images were acquired using the same microscope and camera described above.

### FAIRE procedure.

Whole cells were fixed in growth medium by addition of 37% formaldehyde to a final concentration of 2% formaldehyde for the histone modification mutant experiments, and 1% formaldehyde for the cell cycle experiments, followed by 30 min of incubation at 30 °C. FAIRE results using 1% or 2% formaldehyde were indistinguishable. Formaldehyde-fixed cells in YPD were then quenched with 125 mM glycine for 5 min. The cells were harvested by centrifugation at 1,500 *g* for 5 min and then washed with PBS. The fixed cells were added to lysis buffer (2% Triton X-100, 1% SDS, 100 mM NaCl, 10 mM Tris-Cl [pH 8.0], 1 mM EDTA) and disrupted with glass beads. The extracts were then sonicated (Branson Digital Sonifier) four times each (18% amplitude for 45 s with pulsations of 1 s on and 0.5 s off) with approximately 1 min on ice between sonication sessions. Extracts were then subjected to standard phenol-chloroform extraction [53].

Genomic DNA from each strain used was prepared by glass bead disruption in lysis buffer, sonication, and standard phenol-chloroform extraction. The genomic DNA functioned as a reference in the comparative hybridization to microarrays. For the histone modification mutant experiments, genomic DNA from each wild-type or mutant strain tested by FAIRE was used as a hybridization reference. For all cell cycle experiments, genomic DNA from DBY8724 was used as a hybridization reference.

### DNA amplification and labeling.

Prior to amplification and labeling, aqueous extracts were treated with RNase A and then DNA was ethanol precipitated. DNA yield after phenol-chloroform extraction in FAIRE samples was low, so all samples and references were amplified prior to fluorescent dye incorporation. For all experiments shown in Figure 3, samples and references were amplified using a T7 RNA polymerase–based linear method [54]. Briefly, T7 promoter sequences are ligated to DNA fragments, followed by transcription by T7 RNA polymerase. Using random primers and amino-allyl-dNTPs, the synthesized RNA is reverse transcribed into cDNA and conjugated to either Cy5 or Cy3 monofunctional ester. For the experiments using higher-resolution arrays and the cell cycle experiments, all samples and references were amplified using a random primed PCR-based method [55]. The first amplification round involved use of primer A (59-GTTTCCCAGTCACGATCNNNNNNNNN-39) in conjunction with Sequenase, a modified T7 DNA polymerase. In the second round, primer B (59-GTTTCCCAGTCACGATC-39) was used with Taq DNA polymerase in 25 cycles of PCR. In the final round, the fluorescent nucleotides Cy3-dUTP or Cy5-dUTP were then incorporated directly into the reference and sample in an additional 25 cycles of PCR by again using primer B and Taq DNA polymerase.

### DNA microarray hybridization and scanning.

After labeling, DNA was purified and hybridized to DNA microarrays as previously described [42]. The DNA microarrays were manufactured using a robotic arrayer to print PCR products on poly-L-lysine–coated glass slides as described [32,42,56]. PCR-amplified products represent ORFs, intergenic regions, and other non-coding regions (rDNA, tRNA, transposons, transposon long terminal repeats, telomeres, centromeres, and introns). Generally, each ORF was represented from start codon to stop codon. The intergenic regions consisted of the DNA between annotated ORFs divided such that PCR products were not longer than 1.5 kb, with a few exceptions. The non-coding regions conform to boundaries as annotated by the *Saccharomyces* Genome Database (SGD; http://www.yeastgenome.org) as of the year 2000. Mitochondrial segments did not necessarily conform to annotated functional boundaries. Images were acquired using a GenePix 4000B scanner and Genepix software (Molecular Devices).

### High-resolution oligonucleotide microarrays.

Microarray design is described in Yuan et al. [23]. The microarrays use 50-mer oligonucleotide probes that overlap every 20 bp to tile almost all of Chromosome III and 1 kb of 223 additional regulatory regions. Four microarrays (three biological experiments and one technical replicate) were performed using the wild-type strain BY4741. Data of median of ratios were extracted directly from each array using Genepix. The data were log_2_ transformed and then block normalized as described [23]. Finally, the technical replicates were averaged and treated as one biological replicate, followed by averaging all three biological replicates.

### Data analysis.

Acquired images were inspected visually to remove low-quality spots. Raw data were submitted to the University of North Carolina (UNC) Microarray Database (http://genome.unc.edu). We retrieved the value from each spot as the log_2_ normalized ratio of (median intensity of sample pixels/median intensity of reference pixels), and only the spots with a regression correlation > 0.6 (i.e., those comprised of pixels with consistent ratio values) were downloaded. The log_2_ ratio of each spot was transformed to a *z*-score using the formula, z_x_ = (X − μ) / σ , where X is a retrieved spot value, μ is the mean of all retrieved spots from one array, and σ is the standard deviation of all retrieved spots from that same array. After *z*-score transformation, the mean of all retrieved spots from an array becomes 0, and the standard deviation becomes 1. Following *z*-score transformation, technical replicates from dye-swap experiments were averaged and treated as one biological replicate, followed by averaging all biological replicates.

The data from BY4741 were also used in the comparison to histone ChIP-chip data. For the cell cycle experiments, data were derived from a total of 35 microarrays. Each time point is represented by three or more biological replicates as follows (format: time after release in minutes (# biological replicates, # technical replicates): 0 (4,0), 18 (4,0), 36 (5,1), 54 (4,1), 63 (3,1), 72 (5,1), and 90 (5,1). Classification of promoters into “cycling” or “non-cycling” categories was performed by visual inspection. Readers are free to explore alternate classification schemes.

### Permutation tests.

For Figures 6, 8, and S1, reported *p*-values resulted from non-parametric permutation tests. *p*-Values reported in Figure 6 were derived from 1,000,000 permutations in which labels were randomly reassigned among all unidirectional promoters. The null hypothesis stated that average nucleosome depletion from the tested cell cycle group and time (e.g., G_1_ for time 18) is indistinguishable from all other unidirectional promoters. An empirical *p*-value of 0.05 would indicate that the value obtained from the real data was lower than the value obtained with permuted data 95% of the time. *p*-Values of less than 1 × 10^−6^ indicate that the value obtained from the real data was never lower than the value obtained in the permutations. The *p*-values reported for Figure S1 were derived in the same manner as in Figure 6, except bidirectional promoters were used. For Figure 8, we randomly reassigned each gene to a cell cycle category, using the data for cell cycle–regulated genes only (the number of genes that belong to each category was kept constant). This was done 1,000,000 times. Using the permuted data, for each time point we asked, relative to the observed data, “how often was the difference in FAIRE enrichment between the G2/M promoters and the rest of the promoters higher in the permuted data”? The answer to this question led to the empirical *p*-values reported.

### Data availability.

Raw microarray data are available from the UNC Microarray Database (UMD, https://genome.unc.edu). The data discussed in this publication have been deposited in National Center for Biotechnology Information's Gene Expression Omnibus (GEO; http://www.ncbi.nlm.nih.gov/geo) and are accessible through GEO Series accession number GSE4736. Original microscope images are also available for download (https://genome.unc.edu/pubsup/cellcycle2006/index.html).

## Supporting Information

Figure S1 Nucleosome Occupancy at Bidirectional Cell Cycle–Regulated Promoters Varies with the Cell Cycle Same as Figure 6, but only the bidirectional promoters of cell cycle–regulated genes were analyzed.
(97 KB PDF)

Figure S2 Nucleosome Occupancy at Cell Cycle Gene Promoters (Left) Average profiles for unidirectional and bidirectional promoters of each cell cycle phase group. Solid lines represent average profiles, whereas dotted lines represent the 95% bootstrap confidence intervals. Bootstrapping with replacement was performed to derive 10,000 resamplings of the full sample size. (Right) Profiles of cycling and non-cycling promoters within each cell cycle group.
(113 KB PDF)

Figure S3 Relative Nucleosome Depletion Changes at the Promoters of Cell Cycle Regulators (A–C) *z*-Score values are plotted for the promoters of cell cycle regulators. The promoters were divided into groups (A–C) based on the cell cycle timing of regulation. The error bars are ± standard error of the mean (SEM). Both unidirectional and bidirectional promoters are plotted.
(25 KB PDF)

Figure S4 *CLN3*- and *CLB2*-Responsive Genes Exhibit Large Cell-Cycle Fluctuations in Relative RNA Abundance The relative RNA abundance [30] of G1- and G2/M-regulated genes [30] parsed according to their response to (A) GAL-CLN3 [30] or (B) GAL-CLB2 [30] are plotted. (C) The relationship between nucleosome occupancy change at all unidirectional promoters of cell cycle–regulated genes (*y*-axis moving average, window = 30, step = 1) and RNA level changes for the corresponding downstream gene (*x*-axis moving average, window = 30, step = 1) is plotted for all time points.
(131 KB PDF)

Table S1 List of Promoters Shown in Figure 7
(103 KB XLS)

### Accession Numbers

The UniProt/SwissProt (http://www.ebi.uniprot.org) accession numbers for the genes and gene products discussed in this paper are as follows: *CDC20* (P26309), *CDC28* (P00546), *CLB1* (P24868), *CLB2* (P24869), *CLB5* (P30283), *CLB6* (P32943), *CLN1* (P20437), *CLN2* (P20438), *CLN3* (P13365), *DIG1* (Q03063), *FIG1* (P38224), *FIG2* (P25653), *FKH2* (P41813), *GAL1* (P04385), *GAL10* (P04397), *GCN5* (Q03330), *HAT1* (Q12341), *HAT2* (P39984), *HDA1* (P53973), *HHF1* (P02309), *HHT1* (P61830), *HO* (P09932), *HOS1* (Q12214), *HOS2* (P53096), *HOS3* (Q02959), *HTA1* (P04911), *HTA2* (P04912), *HTB1* (P02293), *HTB2* (P02294), *KAR5/FIG3* (Q04746), *MBP1* (P39678), *MCM1* (P11746), *MSI1* (P13712), *NDD1* (Q08887), *PCL2* (P25693), *PCL9* (Q12477), *PHO5* (P00635), *RAD6* (P06104), *RAD6* (P06104), *RPD3* (P32561), *SEC53* (P07283), *SET1* (P38827), *SET2* (P46995), *SET3* (P36124), *SET4* (P42948), *SET5* (P38890), *SET6* (Q12529), *SIC1* (P38634), *STE12* (P13574), *SWE1* (P32944), *SWI4* (P23302), *SWI5* (P08153), *SWI6* (P09959), *YDR222W* (Q04925), *YKR011C* (Q02209), *YPL056C* (Q02786), *YPR003C* (P53394), and *YPR022C* (Q12139).

## Abbreviations

bp: base pair
ChIP: chromatin immunoprecipitation
FAIRE: formaldehyde-assisted isolation of regulatory elements
kb: kilobase
NFR: nucleosome-free region
ORF: open reading frame
SEM: standard error of the mean

## Note Added in Proof

The article by X. Liu, C. K. Lee, J. A. Granek, N. D. Clarke, and J. D. Lieb, cited in this paper as unpublished data, is now in press [58].
