# Supplementary material for: Cell Cycle–Specified Fluctuation of Nucleosome Occupancy at Gene Promoters
Source: PLoS Genet. 2006 Sep 22;2(9):e158. doi: 10.1371/journal.pgen.0020158 (PMC1570381; doi:10.1371/journal.pgen.0020158)

Figure S1 - Hogan, Lee, and Lieb

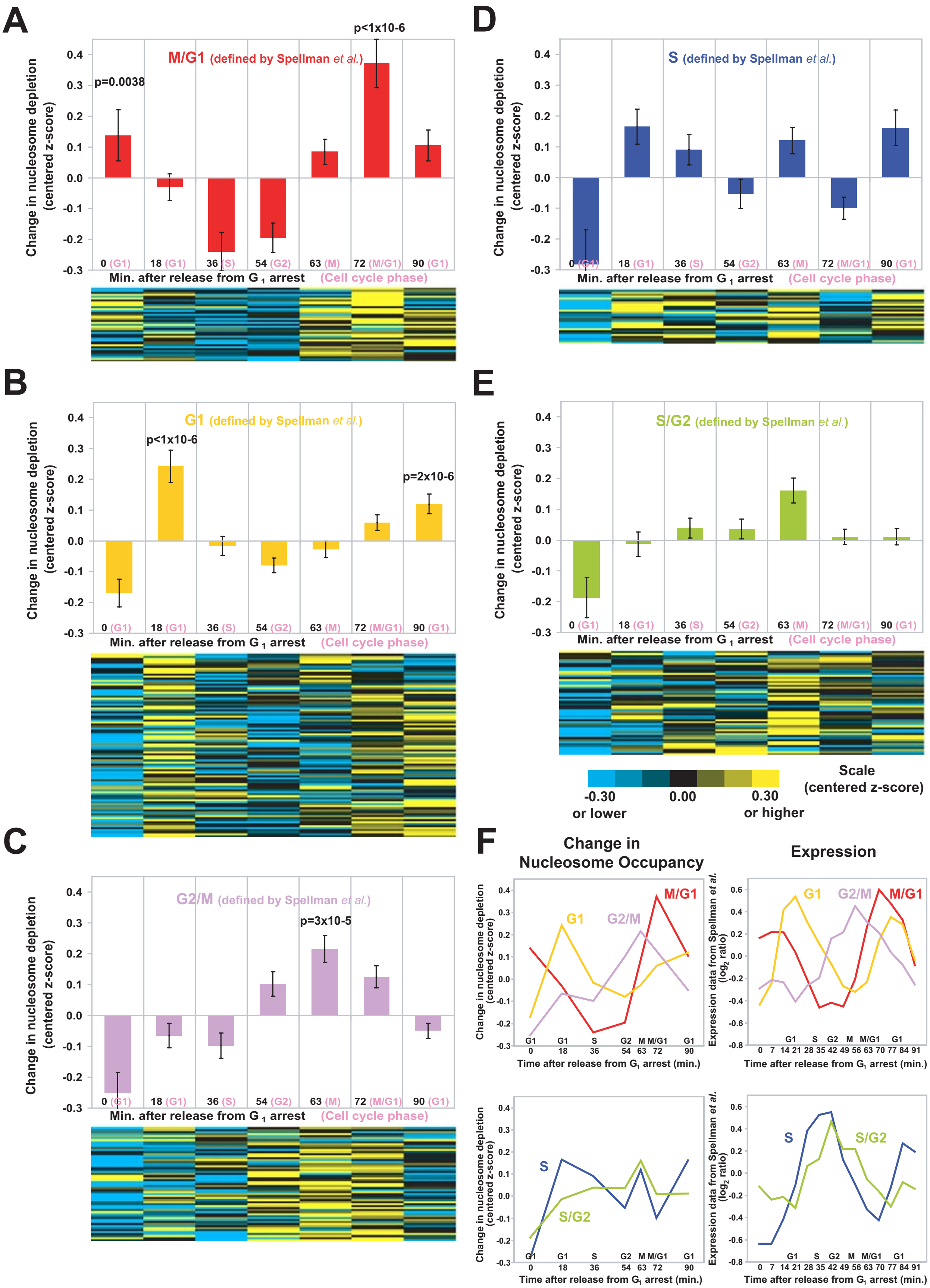

Supplement: Supplementary file 1 [file pgen.0020158.sg001.pdf]
